# Case report: MR-guided laser induced thermal therapy for palliative cingulotomy

**DOI:** 10.3389/fpain.2022.1028424

**Published:** 2022-11-01

**Authors:** Anthony K. Allam, M. Benjamin Larkin, Kalman A. Katlowitz, Ben Shofty, Ashwin Viswanathan

**Affiliations:** ^1^ School of Medicine, Baylor College of Medicine, Houston, TX, United States; ^2^ Department of Neurosurgery, Baylor College of Medicine, Houston, TX, United States; ^3^ Department of Neurosurgery, University of Texas, MD Anderson, Houston, TX, United States

**Keywords:** cancer pain, case report, cingulotomy, laser-Induced thermal therapy, LITT, palliative care

## Abstract

In end-stage cancer, oncologic pain refractory to medical management significantly reduces patients' quality of life. In recent years, ablative surgery has seen a resurgence in treating diffuse and focal cancer pain in terminal patients. The anterior cingulate gyrus has been a key focus as it plays a role in the cognitive and emotional processing of pain. While radiofrequency ablation of the dorsal anterior cingulate is well described for treating cancer pain, MRI-guided laser-induced thermal therapy (LITT) is novel. Our paper describes a patient treated with an MRI-guided LITT therapy of the anterior cingulate gyrus for intractable debilitating pain secondary to terminal metastatic cancer.

## Introduction

Pain is one of the most feared symptoms in cancer, with more than 50% of patients with cancer experiencing pain and more than 33% of those patients experiencing moderate to severe pain (1). Some patients will experience pain refractory to currently available multimodal non-interventional therapies (2). In such cases, lesioning procedures were previously considered. However, with the introduction of intrathecal opiate pumps and neuromodulatory techniques in the 1990s, ablative surgery for chronic pain became less common (3). Nevertheless, in recent years, as these neuromodulatory techniques have proven inadequate for pain relief in certain situations, there has been a renewed interest in neuroablative surgery (3, 4). Surgical approaches of this nature aim to disrupt the pathways responsible for the pathogenesis and transmission of pain. Many regions along the sensory pathway have been identified as surgical targets: the dorsal root ganglion, the spinothalamic tract, the thalamus, and the anterior cingulate cortex, to name only a few (2, 5–11). In particular, the anterior cingulate cortex plays a dominant role in the cognitive and emotional processing of pain rather than the sensory-discriminative aspect (2, 12). This higher order approach makes the cingulotomy particularly suited for patients who experience widespread pain (i.e., pain from head and neck cancers or pain with a significant psychological component) (2).

Traditionally, cingulotomies have been performed using radiofrequency ablation (RFA) or radiosurgery (SRS); however, recently, a third approach has emerged: magnetic resonance (MR) guided laser interstitial therapy (MRgLITT) (3, 13–15). In this approach, laser catheters are used to deliver nonionizing radiation. The absorption of this energy results in subsequent heating and eventual damage to the targeted tissue (3). This technique allows for the use of intra-operative MR imaging, which provides near real-time monitoring of temperature and estimated area of damage to minimize injury to surrounding tissue throughout the ablation (3). Due to technical limitations, no commercially available MRI devices are compatible with current RFA systems, and the treatment effects following SRS are too delayed to be clinically relevant.

There are many examples in the literature that discuss the effective role of anterior cingulotomy in the treatment of chronic pain. But to our knowledge, there have been only a few publications, which include five heterogeneous cases all from a single center, that describe the use of MRI-guided laser induced thermal therapy (MRgLITT) and indicate the safety and feasibility of this indication (13, 16, 17). Of these, only one report mentions the efficacy of the applied intervention (13).

As there is a dearth of information concerning this ablative technique for anterior cingulotomy, we present our experience with MRgLITT to treat severe refractory cancer-related pain in a single patient.

## Illustrative case

The patient was a 21-year-old male with S100+, melanocytic vs. clear cell sarcoma of the left thigh. He was in his usual state of health until early 2021, when he noted back and leg pain with a palpable mass on the left thigh. The original lesion was debulked at an outside hospital in May 2021. Over the next few weeks, he frequently presented himself to local emergency rooms. He was labeled a drug seeker and was repeatedly discharged with small quantities of opioids for pain management. He presented to our hospital in June of 2021 for further evaluation of oncological disease and additional pain management treatment options.

The tumor metastasized further into his right lower extremity, local lymph nodes, axial skeleton, liver, and lung pleura throughout his illness. He received radiation to bilateral femurs and was started on immunotherapy (Ipilimumab, Nivolumab) with mixed response and was next transitioned to the multiple kinase inhibitor, Pazopanib. Despite using these novel therapeutics, the disease progression was rapid, and the pain intensified.

By the time he was referred for neurosurgical pain management considerations, the patient had developed diffuse anasarca and suffered from complications associated with persistent bilateral pleural effusions and hypercalcemia requiring intermittent dialysis. Distant metastases were found at multiple sites throughout the axial skeleton, pelvic, and inguinal lymph nodes that also extended further into the lung parenchyma. Additionally, new areas of enhancement were seen on imaging throughout the lower abdominal, pelvic, and thigh musculature, suggestive of additional metastatic sites vs. multifocal myositis (i.e., radiation recall myositis).

In this particular instance, obtaining adequate pain management proved formidable due to the combined nociceptive, somatic, and neuropathic components. The patient reported the pain with the commonly utilized visual analog scale (VAS). It was consistently rated between 7 and 10 and was not controlled with high doses of orally administered opioids, as daily morphine milligram equivalents (MEE) exceeded 200 mg. Eventually, the patient underwent the implantation of an intrathecal hydromorphone and bupivacaine pump. However, this, too, failed to provide adequate pain relief.

Ultimately, the pain became so severe and uncontrollable necessitating transition to inpatient intensive care unit pain management and surgical drainage of the bilateral pleural effusions. Pharmacologic treatments included low-dose intravenous hydromorphone PCA [1.2 mg every 2 h and 0.6 mg/30 min as needed (prn)], Ketamine (1 mg/kg/hour with boluses prn), and dexmedetomidine (0.8 mcg/kg/hour) infusions, oral acetaminophen (500 mg every 4 h), Methadone [2.5 mg three times daily (TID)], meperidine (25 mg every 2 h prn), Gabapentin (900 mg TID), diazepam (5 mg TID) and Baclofen [5 mg twice daily (BID) and 10 mg nightly], transdermal Clonidine (0.2 mg daily) and lidocaine, as well as Olanzapine, Haldol and Lorazepam as needed for management of pain associated anxiety and delirium. The blood plasma concentration variations of the above polypharmacy pain regimen left the patient fluctuating between periods of lethargy and intractable pain with few remaining treatment options.

After several multidisciplinary discussions with the treating Intensivist, supportive care team, ethics officer, oncologist, and primary care physician, the patient and family elected to proceed with an anterior cingulotomy for palliative treatment of his intractable cancer pain.

### Surgical technique

Similar to the target described by Strauss et al., the dorsal anterior cingulate cortex (dACC) was selected as the ablation target (9). Targets were identified anatomically with available imaging. A single lesion was to be performed within each hemisphere; because of this, we chose a more posterior target area. This lesion was designed to include the cingulate's white matter output while sparing the frontal U fibers.

The patient was placed supine on the operating table in the intraoperative MRI suite. General anesthesia was induced, followed by the administration of pre-operative antibiotics and steroids. The patient's head was placed in the MRI-compatible head holder, and volumetric imaging was obtained.

**Figure 1:**
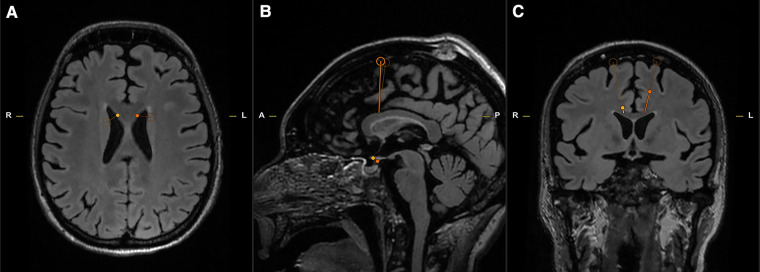
Axial (**A**), Sagittal (**B**), Coronal (**C**) T2 MRI slices for pre-operative stereotactic planning. The open circle indicates the point of entry, and the termination of the thin line marks the target depth (Yellow—Right; Orange – Left).

**Figure 2:**
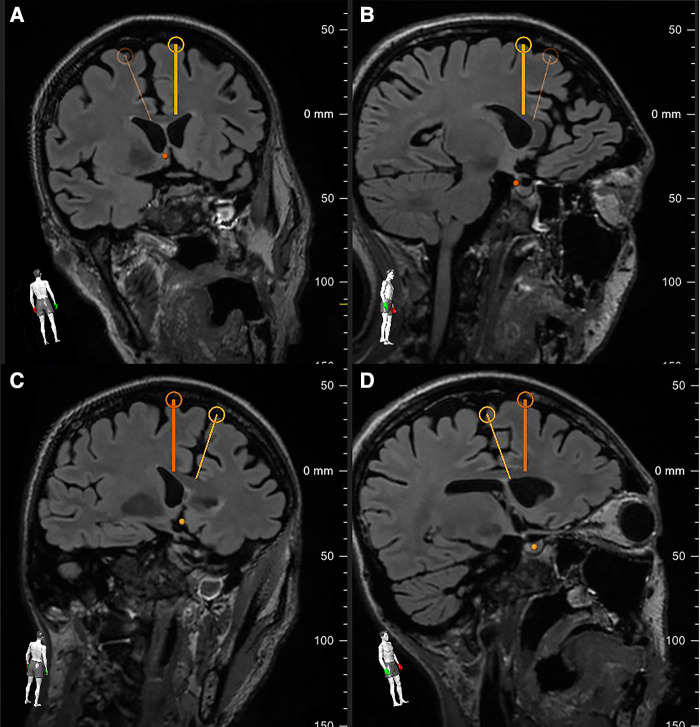
The pre-operative planning software allowed for rotational verification of the right (**A,B** – bold yellow line) and left (**C,D** – bold orange line) trajectories. Note each trajectory's proximity to the white matter tracts intended for ablation while avoiding the associated subcortical U-fibers.

Using the Visualase System (Medtronic), bilateral stereotactic trajectories were planned to the target depth, 24 mm posterior to the tip of the frontal horn, with an entry point through the superior frontal gyrus to avoid blood vessels and maximize the cingulate ablative volume (Figures 1, 2).

**Figure 3:**
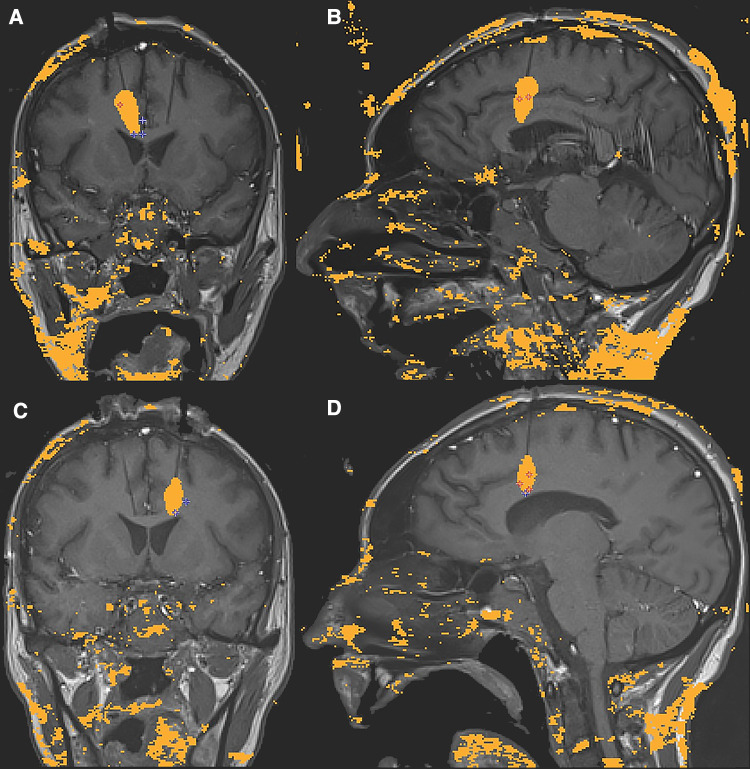
Intraoperative sagittal and coronal MRI thermometry images of the completed right (**A,B**) and left (**C,D**) anterior cingulotomy laser-induced thermal lesions.

**Figure 4:**
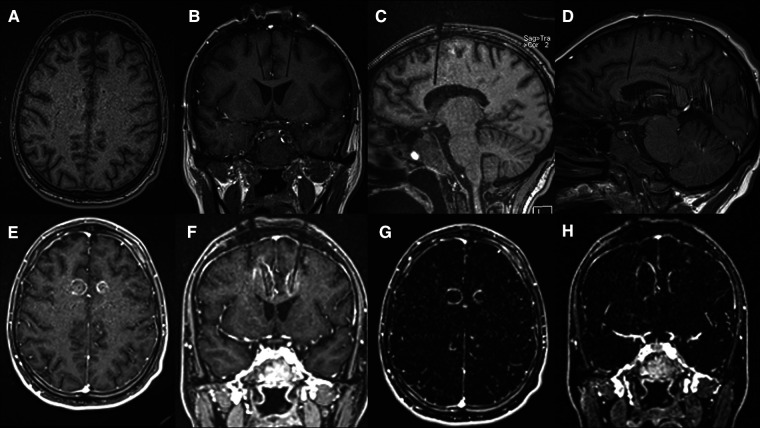
(**A–D**) Pre-ablation T1 sequences obtained for confirmation of the laser catheter location immediately before ablation. Axial (**A**), Coronal (**B**) and Sagittal (**C** – Left and **D** – Right); (**E–H**) Post-ablation T1 + C axial (**E**) and coronal (**F**) images and MRI subtraction, [i.e. (T1 + C)-T1] axial (**G**) and coronal (**H**) slices (**G,H**) demonstrating the area of tissue damage highlighted by surrounding ring enhancement.

A VarioGuide (BrainLAB, Feldkirchen, Germany) was used to align our trajectory to the target. A 3.2 mm drill was used to make the pilot hole for each trajectory. Afterward, a skull bolt was fixated on the skull. Next, the dura was confirmed open with a blunt stylet. If not widely open, Bovie electrocautery may be utilized to ensure the smooth passage of the stylet to avoid catheter deflection during insertion. The blunt stylet is then passed to the pre-measured target depth and removed. Then, a 3 mm laser catheter was inserted through each bolt to depth and secured by sufficiently tightening the bolt-caps. The patient was then transferred to the MRI scanner, where the catheter positions were verified using a T1 sequence. The catheter was retracted manually in small increments from its depth until the intended area was sufficiently ablated. During the ablation, an upper limit of 50-degree Celsius should be set at the superior border of the intended lesion to protect the corpus callosum and U-fibers. The maximum temperature limit for the core of the lesion is set to 90-degree Celsius. Table 1 outlines the specific ablation details for the amount of power delivered and the duration of applied energy. Once the target areas were felt to be sufficiently lesioned (Figure 3), a T1 + C MRI was obtained to confirm that an adequate ablation had been accomplished (Figure 4). Finally, the catheters are removed, and a single interrupted absorbable monofilament suture is used for skin closure of each site.

**Table 1:** MRgLITT anterior cingulotomy ablation details.

Right		Left	
Power (W)	Time (seconds)	Power (W)	Time (seconds)
4.65	31	4.50	20
10.5	24	10.5	16
4.65	28	7.50	12
10.5	66	4.50	23
4.50	28	10.5	19
10.5	59	7.50	28
10.5	102	7.05	7

### Post-operative course

On post-operative day (POD) 1, the patient was minimally reactive and remained intubated and sedated on dexmedetomidine and ketamine infusions. He was clinically noted to require significantly lower doses of narcotics, which allowed for weaning of sedative and opiate infusions. The patient was extubated on POD 2 but remained non-verbal, and on POD 3, the ketamine and dexmedetomidine were discontinued. Within a few days, the patient no longer required intravenous opiates. His regimen consisted of continued intrathecal hydromorphone/bupivacaine, and he was transitioned to an oral regimen of methadone and hydromorphone for maintenance and breakthrough dosing, respectively.

Over the course of the following weeks, the patient's affect remained flat; however, despite this, he was able to be more interactive. Post-operative VAS scores were now being reported as 0–2, in contrast to pre-operative scores, which were routinely 7–10. The patient routinely expressed his gratitude to the treatment team. With his pain more controlled and to the astonishment of other providers, he again began participating in music and physical therapy. More importantly, he was able to have meaningful interactions with family members.

“*For the first time since I met this young man, he was pain-free and remained so for the next three weeks. He was happy, enjoyed food, he continued to engage with PT/OT and confidently declared that he was able to make his own decisions even though his affect had changed since the surgery*”—Primary care physician.

As expected, the patient's disease, unfortunately, continued to progress. Three weeks post-operatively, he developed a massive pleural effusion and sepsis and, as a result, suffered PEA arrest, was unable to be resuscitated, and died.

## Discussion

Pain can be divided into three distinct domains: sensory-discriminative, affective-motivational, and cognitive-evaluative. Treatments that reduce pain frequently target one or more of these areas. Primarily, this means extensive systemic narcotic regimens and neuromodulation *via* intrathecal opioids. Traditional neurosurgical procedures such as percutaneous cordotomy or myelotomy only address the sensory-discriminative aspect of pain by lesioning the pathways from which pain is transmitted from the periphery to the brain (5, 7, 8, 18–21). While this may be effective for certain types of pain. Others are not easily treated, such as facial pain from head and neck cancers, widespread multimodal pain, and pain with a significant psychological component.

Furthermore, the pain relief is often regionally limited and inadequate to treat diffuse multimodal pain. Many chronic pain patients can also develop a psychological component leading to other struggles in their daily activities. Therefore, even if the somatosensory aspect of the pain is controlled, these patients may continue to be burdened by the other detrimental psychological components of their pain.

Cingulotomies have long been performed to treat various conditions, from psychiatric disorders to pain management (2, 14, 22–24). The cingulate gyrus is in the medial part of the cerebral hemisphere, partially occupying the frontal and parietal lobes and wrapping around the corpus callosum superiorly (25). It is separated from the frontal and parietal lobes by the cingulate sulcus and from the corpus callosum by the pericallosal sulcus. It can be broadly divided into the anterior cingulate cortex (ACC) and the posterior cingulate cortex (PCC) through functional, cytoarchitectural, and neural associational differences (26–28). Within these classifications, the ACC can be further subdivided into the dorsal cognitive ACC and rostral affective ACC (26–28). The ACC is intricately connected to various regions throughout the brain, including the premotor and prefrontal regions and other cortical associational areas: striatum, basal ganglia, thalamus, and mesial temporal lobe structures. The mediation of this large neural network *via* the ACC helps explain its contribution to the affective-emotional aspects of pain (12, 25, 29).

Several factors were considered when determining the location of the single lesions within each hemisphere. As mentioned previously, the goal was to create a lesion within the respective cingulate white tracts while maintaining the integrity of the frontal U fibers. One of the advantages of using real-time thermometry during the procedure was our ability to control the lesion size to minimize unwanted side effects. Figure 4 shows that some of the ACC gray matter was not ablated.

Additionally, as the affective component of the pain is reduced post-operatively, it is not uncommon for the patient's overall affect to be similarly impacted. There was a concern that extending the ablation superiorly, above the cingulate white matter tracts, would unnecessarily increase the risk for abulia, a known adverse effect of an anterior cingulotomy. Strauss et al. reported that 4 of 14 patients (30%) developed transient confusion and mild apathy (1–4 weeks) following bilateral stereotactic anterior cingulotomy (9). To avoid early pain recurrence, the vast majority of these patients had a second, more anteriorly placed, lesion made bilaterally. This transient period of depressed affect appears to be unrelated to any procedure-induced callosal damage, which is minimized using the approach described above. It is difficult to determine the long-term significance for patients with increased survival, as this procedure has typically been performed for palliation.

In the above-illustrated case, the goal was to reduce this patient's suffering by disconnecting the outflow from the posterior dACC *via* an MR-guided LITT cingulotomy to dissociate the affective and sensory-discriminative components of his pain. There is no doubting the pre-operative pain experienced by this patient was unbearable to the extent of requiring hospitalization and continuous infusions of sedatives and narcotics until he was in a near coma state for adequate relief. The use of standard pain assessment tools, such as the VAS, is arguably irrelevant in these severe cases. The post-operative pain reduction and ability to dramatically reduce his opiate reliance, resume therapies, and have meaningful interactions with his family are the essential outcomes to take note of in this case.

## Conclusion

Stereotactic anterior cingulotomy can provide palliative relief to well-selected patients suffering from multimodal refractory pain from diffuse metastatic disease by addressing the affective/emotional aspect of pain. The utilization of MRgLITT to perform this procedure is in its infancy. In our limited experience, this technique was well-tolerated in our patient and allowed him to live out the remainder of his life relatively pain-free. Few reports in the literature describe the feasibility and safety of MRgLITT anterior cingulotomy. Continued research is necessary to identify further specific indications and the optimal timing for the procedure to manage intractable cancer-related pain before any broader adoption.

## Data Availability

The original contributions presented in the study are included in the article/Supplementary Material, further inquiries can be directed to the corresponding author/s.
